# Astroglia in Autism Spectrum Disorder

**DOI:** 10.3390/ijms222111544

**Published:** 2021-10-26

**Authors:** Kinga Gzielo, Agnieszka Nikiforuk

**Affiliations:** Maj Institute of Pharmacology, Polish Academy of Sciences, Department of Behavioral Neuroscience and Drug Development, 12 Smętna Street, 31-343 Kraków, Poland; nikifor@if-pan.krakow.pl

**Keywords:** autism, astrocytes, glia

## Abstract

Autism spectrum disorder (ASD) is an umbrella term encompassing several neurodevelopmental disorders such as Asperger syndrome or autism. It is characterised by the occurrence of distinct deficits in social behaviour and communication and repetitive patterns of behaviour. The symptoms may be of different intensity and may vary in types. Risk factors for ASD include disturbed brain homeostasis, genetic predispositions, or inflammation during the prenatal period caused by viruses or bacteria. The number of diagnosed cases is growing, but the main cause and mechanism leading to ASD is still uncertain. Recent findings from animal models and human cases highlight the contribution of glia to the ASD pathophysiology. It is known that glia cells are not only “gluing” neurons together but are key players participating in different processes crucial for proper brain functioning, including neurogenesis, synaptogenesis, inflammation, myelination, proper glutamate processing and many others. Despite the prerequisites for the involvement of glia in the processes related to the onset of autism, there are far too little data regarding the engagement of these cells in the development of ASD.

## 1. Introduction

ASD is a set of neurodevelopmental disorders characterised by reduced sociability, problems with communication, repetitive patterns of behaviour and co-morbid features, including cognitive impairments. The first symptoms of autism appear in early childhood and persist to adulthood, often having a negative impact on everyday life. Although the number of cases of ASD diagnosed in the last decade is increasing [1], there is still no specific answer to the questions of what the main mechanism is and how to treat the symptoms of ASD. The answers are difficult because of the complexity of the symptoms and the variety of factors (gene mutations, environmental events), which may disturb early brain development and increase the risk of ASD. Unfortunately, due to ethical reasons and problems with proper tissue fixation, work with human material is limited. Therefore, to understand the mechanism and accurately reproduce one or more symptoms of ASD, researchers have developed animal models. The majority of cases of autism are idiopathic; here, probably the combination of different environmental and genetic factors interact and lead to ASD development. Because ASD is a complex neurodevelopmental disorder, animal environmental models are usually based on the exposure of pregnant rodents to certain drugs to disturb the normal development of offspring. Another factor linked to the onset of ASD is the activation of the maternal immune system. Gestational inflammation is generally evoked at embryonic day 13 or 15, which induces the most profound symptoms. This is the time period when the neural tube has closed and when progenitors are migrating and proliferating; therefore, this phase of neurodevelopment is highly vulnerable to any environmental insults.

**Figure 1:**
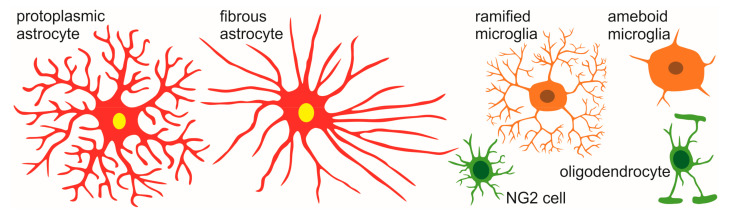
Main types of glia cells in the CNS.

Glial cells support the neuronal net, take part in creating and removing synapses and play a key role in the course of inflammatory processes. All of the types of glia have functions which are essential for proper brain working, protecting neurons from excitotoxicity, buffering ions, cleaning up cellular debris, myelination and many others [2]. Figure 1 depicts the main types of glia cells in the brain.

Changes of astrocytic morphology and number are strictly associated with their response to different pathological processes, such as inflammation and injury, but also normal events present in the brain on a regular basis such as everyday scanning of close environment or changes in a metabolic state [3,4].

There are some clinical reports indicating that glia cells are engaged in pathological processes which may lead to autism. These data relate to a change in the amount of astroglia markers (mainly GFAP) or the number of glial cells rather than a thorough analysis of morphological changes of these cells [5]. The same applies to preclinical models [6,7]. However, substantial heterogeneity exists between studies or even within one study [8,9]. The differences may be due to the difficulty of accessing human tissue and the inadequate processing of the tissue prior to staining. Disruption in glial markers expression in ASD coincides with pathological changes of neurons within brain structures associated with social behaviour, reward, attention or cognitive processes (i.e., prefrontal cortex, hippocampi, striatum, amygdala, cerebellum) [10,11]. Lack of appropriate support from glia cells may be the cause of neuronal or synaptic connection alterations which may lead to the occurrence of neuropsychiatric disorders such as autism.

There is also plenty of evidence for autism-associated brain impairments such as disturbed synaptogenesis, altered number of synapses, imbalance between excitation and inhibition, perturbed neuronal differentiation, improper cortex lamination, and increase and decrease in the amount of parvalbumin-, or calbindin-positive cells in the cortex, striatum and hippocampi in animal models of ASD [12,13].

## 2. Different Models of ASD

The most widely used and validated environmental animal model of ASD is the exposition to valproic acid (VPA). The VPA model is based on epidemiological studies which showed a high incidence of autism in a newborn child following exposure of the pregnant mother to this antiepileptic drug [14]. Furthermore, exposition to VPA results in numerous deficits in social behaviour and communication in rodents [15,16]. These behavioural alterations manifest from the early stages of development [17,18,19].

VPA is a short-chain fatty acid known for its anticonvulsive activity. It has been widely used for epilepsy as well as migraine and bipolar disorder treatment for several decades [20]. VPA has the ability to modulate neurotransmission and regulate GABA transmission and gene expression by inhibition of histone deacetylase activity [21]. Unfortunately, it turned out that VPA is also a potent teratogen leading to vast brain malformations in offspring when administrated during pregnancy. These abnormalities of normal neurodevelopment, in turn, result in developmental delay and autism [22]. Behavioural deficits induced by VPA exposure in humans are also consistently exhibited in rodents.

Another recognised risk factor of the occurrence of psychiatric disorders, besides VPA exposition or genetic mutations, is infection during prenatal life. Activation of the maternal immune system (MIA) caused by viruses or bacteria might lead to neuropsychiatric disorders in the offspring. The MIA model can be divided into three phases. First, viruses or other pathogens activate the maternal immune system and lead to increased levels of proinflammatory cytokines. Consequently, the disturbed integrity of the placental barrier allows for the entrance of maternally derived proinflammatory factors into the fetal circulation. That evokes an inflammatory response in the developing brain of the fetus. Disturbed neurogenesis and central nervous system development will be the cause of neuropsychiatric disorders in the offspring [23].

Due to the nature and specificity of behavioural impairments and structural changes of the brain caused by inflammatory processes, inflammation-based animal models have been widely used to explore certain aspects of human disorders such as autism but also schizophrenia. Moreover, there are plenty of studies and meta-analyses, which have shown that maternal infection increases the risk of the occurrence of neuropsychiatric disorders in children [24]. To study the mechanism behind the association of MIA and ASD occurrence in offspring, pregnant rodents have to be infected with: the lipopolysaccharide (LPS), which leads to a bacteria-like infection; poly (I:C), double-stranded RNA analogue, causing virus-like infection; or influenza virus. However, the chance of developing autism is greater after viral infection, especially when it happens in the first trimester of pregnancy [25]. Poly (I:C) injection to pregnant rodent females is considered to have a stronger construct validity than LPS [23]. Rodents prenatally exposed to poly (I:C) treatment have several socio-communicative features of autism, such as decreased number of social play episodes and deficits in communication, abnormal vocalisation, as well as altered cytokines levels or neuronal density within the brain [13].

About 15% of ASD cases have a genetic component such as coding sequence mutations, chromosomal rearrangements, or copy number variants [26]. For example, Fragile X syndrome is a genetic condition that results in a range of cognitive impairments and is the most common monogenic cause of autism in human patients and animal models [27]. Actually, Fragile Mental Retardation 1 (Fmr1) knock-out (KO) is one of the best preclinical genetic models associated with autistic traits. Altered behaviour in clinical ASD cases and preclinical models is associated with a plethora of functional and morphological changes in the brain. Impairment of synaptic connectivity is a well-known cause of ASD. Fmr1-KO mice exhibit disturbed organisation and morphology of dendrites and synapses, as well as impaired structure of the cortex, cerebellum, and hippocampus, which is known to be a manifestation of improper neuron–glia connectivity.

In addition to the variety of mutations associated with ASD, disturbances of histone 3 lysine 4 (H3K4) methylation during prenatal development may also be the cause of neurodevelopmental disorders, including autism. Lysine-specific demethylase 5C—KDM5C is able to remove methyl groups from tri- and dimethyl H3K4, which have an impact on proper dendritic growth and survival, and development of neurons [28]. Mutation of KDM5C is present in children with Fragile X syndrome. Furthermore, it has been shown that iKdm5C-KO animals have impaired social behaviour, cognitive abnormalities, memory deficits, and enhanced aggressive behaviour [29]. However, recent studies have shown that using some FDA approved drugs such as histone deacetylase inhibitor suberanilohydroxamic acid (SAHA) may force the KDM5C expression and lead to improvement of neuronal maturation and abnormal behaviour [30]. Further studies focused on using SAHA may be very helpful in the therapy of ASD.

In 2017 Pavăl [31] presented the dopamine (DA) theory of autism. He postulated that DA signalling might be involved in the symptomology of ASD. A lot of studies have shown changes in levels of DA and changes of phasic striatal DA events in different brain structures in ASD patients. Moreover, antagonists of dopamine receptors diminished some symptoms of autism (reviewed in [32]). It has been also demonstrated that stimulation of the nigrostriatal pathway using optogenetic tools influences social and repetitive behaviour in mice [33]. Furthermore, mutations of genes encoding dopamine transporters (DAT T356M) lead to hyperlocomotion, reduced social approach and deregulated DA metabolism and synthesis [34] in rodents. García-Domínguez and colleagues [35], in the most recent study, have shown that selective deletion of Caspase-3 gene in the dopaminergic system leads to spatial changes of DA levels, and most importantly, Casp3-deficient mice exhibited impaired social interaction, restrictive interests and repetitive behaviours—the core symptoms of autism. It is worth mentioning that spatio-temporal changes in the dopaminergic system associated with ASD are dynamic; therefore, further research is needed to solve the possible role of DA in ASD.

The models described here are not perfect, but each of them provides valuable information and data needed to understand the mechanism involved in autism. A short list of the chosen animal models of autism is provided in Table 1.

## 3. Symptoms of ASD in Animal Models

### Behaviour Associated with Social Skills

Rodents are social animals with stable social behaviour patterns, such as parental care and social play behaviour [51]. Social play behaviour is a highly rewarding activity, pivotal for the proper development of the brain and social capabilities in mammals. Social deficits found in patients but also in animals are strongly related to the inability to engage in prolonged social interactions with others. Alterations of social behaviour already seen at first postnatal days of animals with modelled autism have a deleterious impact on social interactions in adulthood. This is regarding not only playing with conspecifics but also aggressive and sexual behaviour, which are crucial for survival. The inability of proper engagement in social behaviour in youth results in social isolation and stress in adulthood in both rodents and humans [52].

The first, non-mother directed social behaviour in rats is social play behaviour. It appears for the first time 21 days after birth and peaks between the 28th and 40th postnatal days. Social play events can be socio-positive (e.g., grooming, pinning, pouncing) or rather aggressive (e.g., boxing and pushing away) [53,54]. When rats become sexually mature, some specific playful events disappear, and the behaviour changes into social interactions.

Rats use ultrasonic vocalisations (USVs) to communicate their emotional states with their mother and to establish social contacts with their conspecifics [55,56]. ASD is associated with problems in verbal communication in humans and rodents. What is important is it is now clear that the emission of USVs is not a side effect of locomotion or breathing but has a real purpose. The onset of ASD in humans occurs in early childhood. Identifying autistic-like behaviours in rats in the early period of development is problematic. Therefore, researchers made a useful tool of measuring USVs induced by maternal isolation to described early symptoms of ASD in animals. Neonatal USVs emission differs from calls emitted by adult rats. When separated from their mother and nest, rodents emit USVs with frequencies between 30 and 90 kHz, but mainly about 40 kHz. Later in life, USVs can be divided into two groups based on emotional states of the rodents; calls of the 22 kHz emitted by rats, termed an “alarm” vocalisation, give information about negative emotional state caused by aversive stimuli, aggressive behaviour, stress, and the appearance of a predator [57]. USVs with frequencies above 35 kHz are associated with a positive emotional state; these calls, called “50 kHz”, are emitted inter alia during play. Calls of rodents can also be divided into several subtypes based on their acoustic features [18,58]. Therefore, measuring USVs emission in adolescent and adult rodents may provide further information about the altered social behaviour and communication in ASD models.

Other symptoms of ASD which are documented to be present in animal models, apart from those described above, are the following: (1) second core symptoms: restricted and repetitive behaviours, and (2) co-morbid symptoms: a higher level of anxiety, cognitive impairments, and an increase in locomotor activity.

## 4. Anatomical and Neuronal Changes in ASD

Neurotransmitters play a key role in brain development, memory and behaviour; in general, the proper functioning of the nervous system. Therefore, it is not surprising that ASD is associated with the pathological functioning of the neurotransmitter system. Neurotransmitters most commonly associated with the pathogenesis of ASD are gamma aminobutyric acid (GABA), glutamate, and also, to a lesser extent, serotonin and dopamine [59]. GABA is the main inhibitory transmitter in the brain. It is responsible for proper neuronal migration and maturation during the early development of the CNS. In the mature brain, GABA, together with glutamate, are responsible for proper excitation/inhibition balance. It has been shown that the pathophysiology of GABA transmission in ASD is associated with decreased expression of glutamic acid decarboxylase (GAD65 and GAD67). GAD is an enzyme that catalyses the conversion of glutamate to GABA, and disturbance of this reaction leads to reduced inhibition [60]. Impaired GABA-ergic transmission in ASD may also be associated with a diminished amount of GABA-ergic receptors and inhibitory interneurons [60]. The second main neurotransmitter of the brain is glutamate. Glutamate mediates excitatory signals in the nervous system, regulates synaptogenesis, plays an essential role in learning and memory, and much more [61]. It is important that the E/I balance does not have to be disturbed in the whole brain to lead to ASD. Changes in the levels of glutamate and/or GABA in autistic patients and in preclinical models are rather regional. The study using proton magnetic resonance spectroscopy demonstrated that the level of the main excitatory neurotransmitter is mainly altered in the striatal structures of the autistic brain, in both humans and rodents [62]. The homeostasis of E/I processes in the brain is mainly maintained by astrocytes. Disturbed function of astroglia may result, for example, in the facilitation of excitatory events in the brain [63]. The role of astroglia in controlling of glutamate and GABA distribution in the context of ASD will be described in the next chapter of this review. There are also reports of changes in the dopaminergic and serotonergic systems in autism [31]. Studies have revealed that serotonin or the serotonin transporter levels are altered in autistic children and rodents. Moreover, some selective serotonin reuptake inhibitors have been shown to be helpful in treating some behavioural symptoms of ASD. In addition to serotonin changes, there have been found some fluctuations of the expression of dopaminergic receptors, transporters and dopamine levels in ASD [34,64].

Currently, now that neuroimaging is used to study brains, we know more about brain differences between normally developed people and people with ASD. The impairments in children are already visible at the beginning of growing. The volume of the autistic brain is bigger than of a normal brain, but after few years, the growth, mainly of the frontal lobe, is arrested. Differences in the brain connectivity also appear, which is probably a result of improper white matter maturation [65]. Using functional magnetic resonance imaging, researchers were able to connect structural changes of the brain with behavioural impairments specific for autism. Kim et al. (2015) [66] showed that there are differences in activation of amygdalae between autistic and healthy patients. There are also significant differences between hippocampi and different parts of the cortex—structures associated with memory, communication and social behaviour. There are also some differences in the striatum, which is linked to repetitive behaviour [67]. The changes concern not only the volume or activity of the brain, but also the amount of neurons in the above-described structures as well as the incorrect cortex lamination [10,68].

Impairments of behaviour in clinical cases of ASD and preclinical models of autism spectrum disorder are accompanied by a plethora of functional and morphological changes of the brain. For example, exposure to VPA leads to disturbed gamma-aminobutyric acid signalling and loss of neurons. Moreover, VPA inhibits histone deacetylase which may result in impaired expression of different types of genes or may lead to disturbed DNA repair [69]. Additionally, it may cause alterations in the brain development and synaptic maturation as well as disruption of dopamine and serotonin homeostasis (reviewed in [70]).

### Sex Differences in ASD

Autism spectrum disorder affects males more frequently than females. Despite the lack of a complete understanding of the observed patterns of sex-differential vulnerability to ASD, most of the animal and clinical studies related to ASD have been focused on males. Male sex has been described as a risk of ASD and other neurodevelopmental disorders. However, females are also affected in large numbers, but there is very little empirical data regarding females. The genesis of ASD in both sexes is probably the same but females are thought to be more ”protected” against ASD. However, Braun et al. 2019 [71] showed that females are not really “protected”, they rather have simply different symptoms or anatomical changes of the brain than males. Braun found that female mice with inflammation-based ASD have an increased number of interneurons in the cortex and exhibit anxiety-related behaviour which was absent in males. The group also showed that indeed, some of the symptoms exhibited by males are absent in females; however, contrary to males, females show an elevation in brain inflammation, altered juvenile social behaviour, and a delay in body growth. Because of differences in the manifestation of autistic features, females may be at an elevated risk of missed or late diagnosis. Moreover, it has been shown that woman are better at camouflaging their autism symptoms [72,73], which additionally may mislead doctors and researchers and consequently lead to the wrong diagnosis or conclusions about ASD in females. It is important to remember that exposure to different sex hormones during brain development modulates the formation of neural networks, and it may result in numerous sex differences in the structure and function of the brain. These differences are likely to be seen in the disparate gender susceptibility to neuropsychiatric diseases. Therefore, research on both sexes is crucial to understand the mechanism of the brain disease development.

## 5. Astrocytes as Important Players in the CNS

Astrocytes are the main cells responsible for maintaining brain homeostasis. They are also involved in mechanisms associated with the metabolism and signalling between other cells in the central nervous system such as neurons, oligodendrocytes and microglia. To maintain these functions, astrocytes express a lot of different molecules and receptors. Moreover, astroglia have a plethora of different transporters and pumps, which are essential for proper signalling between the brain cells. In opposition to neurons, astrocytes are not electrically excitable, so they “talk” using the fluctuation of different ions such as calcium, sodium and potassium. This intracellular transport of the ions is possible thanks to the integration of the astrocytes into the syncytia in which the astroglial membranes are connected by the gap junctions (small channels formed by the connexin (Cx) protein family, particularly Cx30 and Cx43).

**Figure 2:**
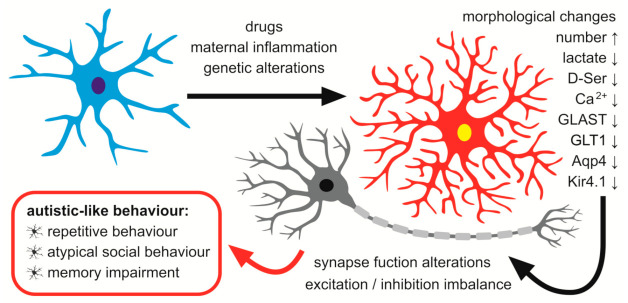
The possible role of astrocytes in the development of autism. Genetic mutations, prenatal exposition on some drugs and prenatal inflammation affect astrocytic morphology and functions. It may result in alterations of synapse functions, imbalanced homeostasis between excitation and inhibition and dysregulation of the nervous system. This, in turn, may lead to autistic-like behavioural changes. D-Ser: D-serine; GLAST, GLT-1: glutamate transporters; Aqp4: aquaporine 4; Kir4.1: inward rectifying K^+^ channels.

Ion homeostasis is a critical function of astroglia. Astrocytes regulate potassium ions and water flow in the extracellular space and between neuronal cells using inward rectifying K^+^ channels (Kir) for buffering K^+^ and water channels—Aquaporin4 (Aqp4)—needed for clearing the cellular debris by the glymphatic system [74]. Altered homeostasis of astrocytic water channels and K^+^ ions in the brain lead to altered balance between neuronal excitation and inhibition. Even small alterations in extracellular K^+^ concentration may produce hyperexcitability of neurons. Reduced Kir4.1 expression (but not other K^+^ channels) increases extracellular K^+^. Additionally, conditional knock-out of Kir4.1 depolarises glial membranes and inhibits potassium and glutamate uptake [75]. Furthermore, it has been shown that loss of water channels such asAqp4 present on astrocytes may lead to an impaired K^+^ buffering and consequently to increased excitation of neurons which underlies many neuropsychiatric disorders, including autism. The scheme depicting alterations in astroglia–neuron interactions (along with their probable causes) that possibly contribute to the development of autism is shown in Figure 2.

It has been known for a long time that astrocytes are essential for maintaining proper brain activity. Blocking astrocytic metabolism by fluorocitrate leads to seizures and may be the cause of obsessive/compulsive behaviour in rodents [76]. Astrocytes are responsible for the clearance and transport of glutamate, which is possible due to the presence of glutamate transport proteins GLAST and GLT-1 on astrocytic membranes. Abnormalities of glial cells regarding glutamate metabolism may lead to behavioural impairments in animals. Aida and colleagues (2015) [77] found that GLT-1 deficiency increases neuronal excitation and leads to excessive repeated behaviour, including self-injuring in mice. That should not be very surprising knowing that GLT-1 accounts for more than 90% of glutamate uptake in the brain. These findings were recently confirmed by another research group [78,79]. Furthermore, postnatal disturbance of glia proliferation may lead to hyperactive behaviour in open field tests and alterations of social interactions in rats [80]. In addition to the abovementioned glutamate transporters, the amino acid neurotransmitter cycle is also supported by glutamine synthetase (GS). GS converts glutamate into glutamine which will then be used by neurons. As it turns out, GABA-ergic neurons are more dependent on astrocyte glutamine and thus more sensitive to decreased GS activity than excitatory neurons, so lack of the astrocytic GS may lead to the altered functioning of the inhibitory neurons. Furthermore, inhibiting the astrocytic GABA_B_-Gi pathway in the striatum leads to the enhanced attention in mice and reduces behavioural hyperactivity in mice [81].

There is still growing evidence that not only neurons but also astrocytes are involved in memory formation. This is possible due to the release of the astrocytic transmitters such as D-serine and ATP following the activation of astroglia [82]. Astroglia may contribute to cognition processes not only by modulating synapses but also by activating the neighbored neurons by supplying them with lactate. Since the last decade, the pivotal role of astrocytic lactate in memory formation and learning has consequently been confirmed. Moreover, it has been shown that the lack of lactate transportation may even result in amnesia in rodents (reviewed in [83]).

While there are indications that astrocytes underlie the mechanisms leading to psychiatric disorders such as autism, and their altered functioning may lead to behavioural changes in rodents, there is still little research on this topic, especially regarding social behaviour and communication, which should be the next step in understanding the mystery of cooperation between neurons and astrocytes.

### Shape Changes of Astroglia

Astrocytic processes tend to change their morphology in response to CNS inflammation, epilepsy, injuries, and neurodegenerative disease as well as during the normal, non-pathological interactions with neurons. These morphological changes of astroglial silhouettes may act as excellent indicators of the current state of the brain; a detailed morphological analysis is able to show changes already in their initial stages. Most astroglia is generated by the 16th postnatal day [84]. However, through this period of time, they tend to change their morphology from immature, characterised by a simpler silhouette with quite short, uncomplicated processes, to mature, characterised by a fine, largely ramified profile. At postnatal day 14, all astrocytes should be characterised by almost-mature morphology, even though they do not reach full maturity until postnatal day 30 [85]. This process of astrocytic maturation is temporally correlated with the maturation of the synapses and the expression of some glutamate receptor subunits [86], as well as their identity may be overwritten by surrounding neurons [87]. During synaptogenesis, astrocytes’ morphology becomes more complex, with small but very important perisynaptic processes which are engaged in establishing “tripartite synapses” with neurons. Astrocytic perisynaptic processes are very rich in a plethora of receptors, adhesion molecules and ion channels which are crucial for maintaining functions of synapses (reviewed in [88]).

Astrocytes, based on their morphology, can be divided into two main sub-types, namely, protoplasmatic and fibrous. Fibrous astrocytes are distributed in white matter and have an elongated shape with numerous, long processes. Protoplasmatic astrocytes reside in grey matter and they are often called bushy because of their shape and highly ramified processes. The processes are sandwiched between neurons for the purpose of being in close contact with synapses. Recent studies have shown that protoplasmatic astrocytes are diverse and manifest layer-specific molecular and morphological differences. Lanjakorsiripan et al. (2018) [89] demonstrated that astrocytes in mice cortex have different shapes in different cortical layers. For example, astrocytes from layer II/III tend to elongate radially in contrast to astroglia in layer VI, which elongate tangentially. Moreover, using 3D Sholl analysis, they found that astrocytes in layer II/III have more intersections than layer VI astrocytes. The shape of astrocytic silhouette turned out to be associated with their function in the cortex. Although most of the genes expressed on astrocytes are similar, some of these genes, mainly engaged in synaptogenesis and metabolism, were enriched in astroglia in the upper cortical layers (*Dio2, Mertk, Slc1a3*). Genes highly expressed in deeper layers were mostly associated with proliferation (*Cxcr7*) or astrocyte–neuron communication (*Gfap*). Furthermore, the Lanjakorsiripan group also found that the formation of layer-specific characteristics of cortical astrocytes is strongly dependant on neurons. Without neuronal signalling astrocytes failed to form the laminar organisation. There is a strong connection between astroglia and neurons, and they should not be studied separately but rather together as one coexisting/symbiotic net.

Unfortunately, there is little precise research on the shape of astrocytes in ASD. This is quite surprising because there are available tools to perform the detailed analysis of cell morphology. Using ImageJ software [90], different research groups were able to distinguish between different types of glial cells, including all intermediate states of the cell [91]. Parameters which can be used to quantify morphological changes of glia can be associated with a number of processes (sum of intersections, maximal number of intersections, ramification index (obtained with Sholl analysis)). It is also possible to describe the size and shape of the cell (convexity, solidity, etc.). Moreover, using fractal dimension and lacunarity we are able to study the complexity of the cell. Since astrocytes are very heterogeneous plastic cells and they can change their morphological and functional properties throughout their lifetime, it seems important to have a closer look at the morphological changes in astrocytes in further research. Nevertheless, it should be taken into account that the change in the shape of astrocytes and the gain or loss of some protective or harmful functions may occur simultaneously. The impact of these changes on the course of various processes/diseases will change depending on the balance between the protective and harmful alterations that have occurred [92].

## 6. Astrocytes in ASD—Insights from the Clinical Cases and Preclinical Models

Disturbances in the process of gliogenesis may have an impact on proper brain functioning. Abnormalities in both synaptogenesis and balance between excitation and inhibition in the brain are thought to constitute the mechanisms of development of various neuropsychiatric disorders, including autism. Evidence for engagement of astrocytes in ASD onset come from clinical and preclinical studies. Codagnone and colleagues (2015) showed that VPA exposed rats, sacrificed at postnatal day 35, have increased glial fibrillary acid protein (GFAP) immunostaining levels in the medial prefrontal cortex and hippocampus, especially in the CA3 subfield. Despite increased GFAP immunoreactivity, which is often associated with pathological processes in the CNS, there are some studies showing changes in the number of GFAP positive cells in VPA and poly (I:C) models [93,94,95,96], but studies regarding the morphological analysis of glia silhouette in autism are limited [11,13]. Given the still growing knowledge about the pivotal role of those “shape changes” for proper neuronal functioning, a lack of profound morphological analysis of these cells in ASD is quite surprising. A lot of clinical studies using different techniques (ELISA, PCR, microarrays) have also found signs of glia activation in different structures of ASD-affected brains [10,68,97]. Moreover, these alterations are found even in adult brains, which suggests that gliosis-like changes and neuroinflammation may be a permanent state of the central nervous system of ASD patients.

Inflammatory processes during the vulnerable period of neuro and gliogenesis may have deleterious consequences on the proper development of the brain. In response to the influx of cytokines, astroglia (and microglia) start to proliferate and change their shape. Depending on the size of the gliosis, glia may start to recruit leukocytes and contribute to the tissue damage [98]. The escalating gliosis may alter the main function of glia cells associated with synaptogenesis and functioning of developing synapses, which consequently could lead to improper working of the neuronal net and brain disorders. The ASD-phenotype in the aforementioned MIA models may be caused by altered levels of cytokines, especially IL-6, TNFα or IL1β. In this case, crucial for the ASD onset seems to be the disrupted permeability of the blood brain barrier (BBB), which is partially formed by astroglia [99]. The BBB integrity is essential for preventing neuroinflammation in the CNS. Moreover, astrocytes may further increase brain damage caused by inflammation through proinflammatory signalling; however, ironically, at the same time glia scar also protects the brain from overpowered inflammation [100].

### 6.1. Astrocytes and Metabolism

Systemic inflammation found throughout the body of ASD patients can be triggered by many factors, including genetics, age, gender, diet, environmental exposures, drugs and also the gut microbiome. Recently, the microbiome is of particular interest in the context of ASD onset. The occurrence of ASD in children is often accompanied by gastrointestinal distress (reviewed in [101]). Over the past two decades, preclinical and clinical studies show the directional crosstalk between the gut microbiota and the brain, known as the microbiota–gut–brain axis [102]. The gut microbiota may directly influence the brain through the vagus nerve. The microbiome may also modulate the activity of the enteric nervous system by releasing GABA, short-chain fatty acids, which may regulate the levels of other neurotransmitters such as brain-derived neurotrophic factor or serotonin [103]. It has been demonstrated that metabolites of *Lactobacillus reuteri* (the component of the microbiome) may increase the oxytocin level and diminish some of the behavioural symptoms of ASD [104]. The gut microbiota has been shown to regulate microglia function in mice [105]. Moreover, the gut microbiota may metabolize dietary tryptophan into aryl hydrocarbon receptor (AHR) agonists. Those AHR agonists have been shown to activate astrocytic AHR signalling and abolish inflammatory processes in the CNS [106].

The alteration of the permeability of intestinal epithelia caused by altered gut microbiome composition leads to the influx of the inflammatory factors to the blood stream, leading to systemic inflammation. In turn, the ongoing inflammatory processes may alter the integrity of the BBB, and subsequently cause neuroinflammation [107]. Proper composition of the microbiome may influence the course of the ongoing inflammation by release of butyrate, which may diminish the amount of the proinflammatory cytokines. Butyrate itself can alter synaptic transmission, spontaneous neuron firing and glutamate release in cultured cells, as well as facilitate GABA release [108], which clearly has an influence on the excitation/inhibition of homeostasis often disturbed in ASD. Moreover, decreases in butyrate may lead to the disruption of BBB integrity. It has been also shown that switching from glucose to alternative substrates for the Krebs cycle, such as butyrate, has neuroprotective effects [109,110]. Furthermore, astroglia are capable of fatty acid oxidation, a function probably exclusive to these cells in the CNS. In addition, astrocytes, through their specific receptors, may also respond to the local level of nutrients and then switch from using glucose to ketones [111]. Of additional importance are genes engaged in ASD which are enriched only in astrocytes and are associated with metabolism: 4-aminobutyrate aminotransferase [112], fatty-acid-binding protein 7 [113] or glutathione S-transferase 1 [114]. Furthermore, treatment with the ketogenic diet has been shown to change the ramification of the astrocytes [4], indicating the engagement of glial cells in the brain metabolism.

The aforementioned data open a new route for searching for new therapies for ASD and other neurodevelopmental diseases. Actually, the first data regarding the use of different diets in ASD are already published. Studies have shown the improving effect of the ketogenic diet on behavioural symptoms in autistic children [115]. Similar conclusions are drawn from experiments on VPA exposed rodents, as well in BTBR and Mecp2 mice.

### 6.2. Impaired Calcium Signalling in Astrocytes—Influence on ASD

Astrocytic activation is manifested by an increase of Ca^2+^ mainly mediated by type 2 1,4,5—tripsphosphate receptors (IP_3_R2). Interestingly, the IP_3_R2 gene has been found to be affected in ASD patients [116]. However, the role of astrocytes in ASD onset is still unclear. One of the crucial roles of astroglia is maintaining the proper formation of synapses. Wang and colleagues (Wang et al., 2021) have shown that astrocyte-specific IP3R2 conditional knock-out mice (Aldh1L1—CreER:IP3R2^loxp/loxp^) have atypical social and repetitive behaviour. Furthermore, to find the neurobiological mechanisms underlying the autistic features present in IP3R2 mutant mice and astrocyte-specific knock-out mice, Wang et al. (2021) [117], using in vivo microdialysis, analysed the levels of gliotransmitters in the medial prefrontal cortex, which is engaged in social behaviour in rodents. They found remarkably lower levels of ATP in both of the knock-out mice. Moreover, ATP treatment mediated by astrocytic P2X2 receptors rescued the social behaviours in mice and facilitated the GABA-ergic transmission. Findings from this study have shown the potential role for astrocytes in ASD pathogenesis, as well as identified ATP as a new potential molecular player in ASD, thus confirming the synaptopathy hypothesis of autism.

A study by Guerra-Gomes et al. (2018) [118] showed that disruption of the astrocytic mechanism of Ca^2+^ release from the IP_3_R is linked with worsening of cognitive function, and that they are crucial for the functional and structural integrity of neuronal circuits in the prefrontal cortex. There is also plenty of evidence from researches on IP_3_R2-KO mice, which have demonstrated the pivotal role of astrocytic Ca^2+^ signalling in neuron-dependant cholinergic modulation of cortical and hippocampal plasticity (reviewed in [119]). Abnormalities of cholinergic projection and receptors have been found in the brains of autistic people. Given the poor outcomes of many patients affected by ASD, the need for a novel and more effective therapeutic strategy is critical. Moreover, deletion of the gene coding the α7 nicotinic acetylcholine receptors (α7-nAChRs) is associated with the development of symptoms of autism [120]. Increasing literature data support the therapeutic search for selectively targeted cholinergic drug interventions in the treatment of ASD. The compounds binding to the α7-nAChRs seem to be particularly important here. Positive allosteric modulators (PAM) and their ability to antagonise agonist-induced desensitisation make them better than the “usual” orthosteric agonists. Additionally, there are still evolving data regarding a possible metabotropic-like function in membrane depolarisation of these ionotropic receptors. The metabotropic mechanisms of α7-nAChRs depend on the release of Ca^2+^ from intracellular (mainly from the endoplasmatic reticulum) stores by engaging G-proteins and second messengers such as IP_3_. The mobilisation of Ca^2+^ from their intracellular stores is not only limited to neurons. Sharma et al. (2001) [121] showed that activation of astrocytic α7-nAChR led to an increase of intracellular Ca^2+^ concentration by activating calcium-induced calcium release (CICR). As already mentioned, the imbalance between excitatory and inhibitory processes in the brain is a possible cause of ASD occurrence. Immunocytochemical studies have shown diminished levels of GABA receptors, the number of inhibitory interneurons and enzymes responsible for proper glutamate/GABA metabolism in α7-nAChR knock-outs. What is worth mentioning is that there are data that show that the activation of not only neuronal but also glial α7-nAChR results in decreased inflammation and neurotoxic oxidative stress [122]. The data also suggest that administration of α7 selective type PAMs (PNU-282987) may stimulate GABA-ergic inhibitory action and hyperpolarise pyramidal neurons in CA3 of the hippocampus and restore disrupted hippocampal auditory sensory gating [123]. Therapeutic approaches targeting α7-nAChR not only modulate GABA-ergic transmission but also have an impact on the course of inflammatory processes, which are known to be one of the causes of the occurrence of autistic features in human and animal models. In MIA models of autism, modulation of cholinergic transmission, especially by agonists of α7-nAChR [124], results in diminished levels of proinflammatory cytokines such as IL-6 and reduced anxiety behaviour in MIA offspring. Furthermore, the loss of α7-nAChR in the offspring increases their vulnerability to MIA-induced autistic symptoms. However, the role of α7-nAChR modulators in adults and the contribution of glial cells of autistic-like animals in cholinergic modulation is unknown. The evidence from studies on α7-nAChR showed that its agonists increase the density of astrocytes, and moreover, the neuroprotective role of PNU-282987 was only seen when neurons were cocultured with astroglia [125]. Without astrocytes, the viability of neurons was decreased. This neuroprotective effect could be caused by an enhanced release of anti-inflammatory TGFb and anti-excitotoxic glutamine synthetase (GS) by astrocytes stimulated by PNU administration.

## 7. Other Glial Cells in ASD

### 7.1. Microglia

As written above, astrocytes are crucial for maintaining the brain function; notwithstanding, microglia, rather than astrocytes, constitute the major component of the immune system in the brain. In a normal state, microglia study their environment for any signs of damage through a continuous reconstruction of finely branched processes. Retraction of microglial processes becoming macrophages capable of phagocytosis are signs of the pathological state of the CNS [126]. First, microglial cells appear at embryonic day 8. They are mainly clustered near the hippocampi and corpus callosum of the rodent brain. Similar to astrocytes, microglia also mature during the first two to three postnatal weeks. In the beginning, microglia are rather ameboid in shape, but at about postnatal day 30, they become ramified and ready to inspect their close environment. Infection, or other factors with the ability to disturb neurogenesis, may impair the maturation of microglia. This can be manifested by an increased ratio of ramified (mature) to ameboid (immature) microglial cells and might have consequences for the brain connectivity and behaviour [127,128]. Activated microglial cells release different neurotrophic factors influencing the neuronal survival, such as nerve growth factor, neurotrophins, glial-derived neurotrophic factor, interleukins, and many more. On the other hand, microglia-derived factors also include proinflammatory mediators such as IL-6, tumor necrosis factor (TNF), or nitric oxide (NO), which are associated with neurotoxicity. Types of factors released by microglia strongly depend on the kind of pathological state of the brain [129]. Another crucial role of microglia is a process of synapse removing, called “synapse stripping” or “pruning”. From recent studies done by Weinhard et al. (2018) [130] it is known that microglia may use trogocytosis to check and then phagocyte abnormal/unnecessary synapses.

This engagement in inflammatory processes and synapse pruning of microglia seems to be very important in the context of autism occurrence because both inflammation and disrupted synaptogenesis underlie the occurrence of autistic symptoms. Such as in the case of astrocytes, there are findings from preclinical and human studies showing the alterations in the functioning of microglia in ASD [11,13,131,132,133]. The impairments in human brains regard: increased density of microglia, abnormalities in their morphology, elevated expression of IL6 and TNF, genes related to the activation of microglia, and up- or downregulation of complement receptor 3, which is engaged in a proper synaptic engulfment. Signs of inflammation in the human ASD brain visible on MRI scans have also been reported (reviewed in [134]). Similar signs are also present in animal studies [95,135,136]. Interestingly, the greater proportion of autism in males may also be due to the difference in microglia maturation during the brain development in both sexes. The so-called microglial development index (MDI), based on global gene expression patterns, tends to be higher in females than males during early development and early adulthood [127]. Consequently, the immune system’s response to proinflammatory factors in females and males may also vary, thereby increasing/decreasing the risk of developing symptoms of autism. Reduced number of chemokine receptor Cx3cr1 (microglial-specific chemokine receptor) leads to reduced synapse pruning, deficits in social behaviour and increased repetitive patterns of behaviour in mice. The decreased number of Cx3cr1 receptors was found only in male but not in female rodents with inflammation-based ASD (reviewed in [137]). In a pilot study, Chavez-Valdez et al. (2019, [95]) showed that activation of Toll-like receptors 3, which are present mainly on glia cells, during the neonatal period produced dimorphic response with early activation of apoptotic pathways in female mice and necrosis in males. Poly I:C injection in this study also led to activation of inflammatory cascades in both sexes, which then persists in female mice. Moreover, microglia activation was attenuated in females in comparison to males and persisted until 7 days after the Poly I:C injection. The group of Chavez-Valdez did not observe sex-specific patterns regarding astroglia activation. There are also sex differences in reaction to proinflammatory substances such as LPS. LPS exposition led to greater reaction and accelerated transcriptional maturation of male microglia [138].

### 7.2. Oligodendroglia and Their Precursors

Oligodendrocytes are the next type of glia in the CNS. Their main and most important role is making the myelin sheaths which are pivotal for the proper propagation of action potentials through the axons. Myelin increases the capacitance of the neuronal cell membranes and allows to increase the speed of impulses. The second type of oligodendroglia-like cells are polydendrocytes, or precursors of oligodendrocytes (OPCs). These cells also express the NG2 protein; therefore, they are referred as to NG2-cells. NG2 is a transmembrane proteoglycan containing chondroitin sulfate. NG2 was thoroughly described in the late 1970s when it was discovered that this proteoglycan is needed for the differentiation of glial and nerve [139]. The NG2 protein can react with both extracellular matrix proteins and the actin cytoskeleton, and thus participate in cell migration and proliferation. NG2 also may participate in integrin-independent cell adhesion [140].This suggests that NG2 cells may be involved in cell–cell and cell–protein interactions, which may be related to proliferation, migration, cell adhesion and stabilisation of the entire structure of the neural network [141]. Polydendrocytes are involved not only in the development of the brain. In the mature brain, their processes surround Ranvier’s constrictions in the myelinated axons, and neurons form fully functional synapses with NG2 cells [142].

Increasing evidence links neuropsychiatric conditions with white matter alterations. Some studies have shown that ASD may be associated with altered white matter integrity and changed myelin thickness, especially in the corpus callosum of ASD patients [143]. The changes of the white matter have also been shown in animal models. An association between myelination of the medial prefrontal cortex and deficits in social behaviour in mice has been demonstrated [144]. Moreover, Graciarena et al. (2019) [145] found an alteration in the number of oligodendrocytes and oligodendrocyte precursors in mice exposed to VPA. Myelin is produced by oligodendrocytes during development but also in adult life, and recently, it has been reported that myelin actively participates in the modulation of speed and synchronicity of action potentials [146]. Moreover, oligodendrocyte progenitor cells are synaptically innervated by neurons throughout the CNS [147]. The changes in this “communication link” between neurons and oligodendroglia or/and oligodendroglia precursors may play a key role in the neuronal circuit disruptions that occur in neuropsychiatric diseases such as ASD. The genetic model of ASD, the BTBR T^+^ Itpr3^tf^/J mice, recapitulates the main symptoms of ASD (deficits in social behaviours, repetitive behaviours and unusual vocalisation) [148,149], and Stephenson and colleagues (2011) [150] found that BTBR mice have increased immunoreactivity of NG2 cells. Moreover, the enhanced immunoreactivity of these cells coincided with alterations of their size. The findings described here may indicate the possible role of oligodendroglial cells and their precursors in ASD pathogenesis.

## 8. Conclusions

Experimental data regarding the role of inflammatory processes, disturbed synaptogenesis and neurogenesis in the occurrence of autism spectrum disorder allow to hypothesise that disruption of normal glia function, which are the main players in exactly these processes, may underlie the occurrence of ASD or contribute to worsening or/and improvement of autistic symptoms. Astroglia cells are in intimate contact with neurons, and any changes of the close environment of those cells lead to dynamic morphological changes of their shape. Moreover, alterations of morphological features of glial cells are strictly associated with their function in the central nervous system (CNS) [151,152]. There are some data from animal and human studies concerning the role of astrocytes in ASD; however, the conclusions are usually based on superficial analyses of glia markers’ levels (usually GFAP). Given the still growing knowledge about the pivotal role of those “shape changes” for proper neuronal functioning, a lack of profound morphological analysis of these cells in ASD is surprising. Many studies focus only on the functioning of neuronal networks, and despite the prerequisites for the involvement of glia in the pathology of autism, such as increased expression of glia-associated proteins in the brains of autistic people and animals, or disrupted homeostasis of E/I balanced caused by alterations of astroglia functions, there is still little research on astrocytes in context of ASD.

In summary, reported signs of astroglial, but also oligodendroglial and microglial dysfunction in the autistic brain indicate that gliopathology may be a reason for ASD occurrence. Thus, not only neurons but astroglia and other glia cells may represent presumptive targets for novel therapeutic strategies. Nonetheless, despite evidence for a potential role of glia in ASD, the researchers are still mainly focused on basic parameters associated with glia activation, such as GFAP levels, immunoreactivity or number of cells. In the future, we should focus more on the astroglial cells and their role in neuropsychiatric disorders, especially in the context of mutual interactions between neurons and astrocytes.

## Figures and Tables

**Table 1:** A short list of the chosen animal models of autism.

Models	Basis	Mechanism	Observed Behavioral Alterations	Bibliography
LPS	inflammation	increased levels of proinflammatory cytokines in the brain caused by bacterial infection	disturbed communication, altered social behaviour, increased repetitive behaviour	[36]
POLY (I:C)	inflammation	increased levels of proinflammatory cytokines in the brain caused by viral-like infection	disturbed communication, altered social behaviour, increased repetitive behaviour	[19,37]
VPA	chemical pharmacological	a potent teratogen leading to vast brain malformations, inhibition of histone deacetylase, GABAergic signalling disruption	disturbed communication, altered social behaviour, increased repetitive behaviour, anxiety	[16,38]
PPA	inflammation	dietary and gastrointestinal agent initiating neuroinflammation and gliosis	altered social behaviour and cognition	[39]
FMR1	genetic	mGluR5 hyperactivation	disturbed communication, altered social behaviour, seizures, cognitive impairments	[40]
SHANK1	genetic	disruption in synaptic transmission	disturbed motor function, memory, altered communication, no changes in social interactions	[41,42]
SHANK3	genetic	disruption in synaptic transmission	disturbed communication, altered social behaviour, altered learning and memory	[43]
TSC1, TSC2	genetic	altered mTOR signalling	altered communication, altered memory and learning	[44]
NLGN1,2,3,4	genetic	disturbed synaptogenesis	increased stereotypic behaviour, altered sociability	[45]
RELN	genetic	disturbed neuronal positioning and synaptogenesis	disturbed communication, altered social behaviour	[46]
CNTNAP2	genetic	decreased glutamate receptor expression and transmission	disturbed communication, altered social behaviour, hyperactivity	[47]
CACNA1C	genetic	disruption in synaptic transmission	disturbed communication, altered social behaviour	[48]
GABRB3	genetic	altered GABAergic signalling	disturbed communication, altered social behaviour, hyperactivity, altered cognition	[49]
UBE3A	genetic	disruption in synaptic transmission	disturbed communication, altered social behaviour, seizures, altered cognition	[50]
